# Sex Differences in Adverse Liver and Nonliver Outcomes in Steatotic Liver Disease

**DOI:** 10.1001/jamanetworkopen.2024.48946

**Published:** 2024-12-04

**Authors:** Taotao Yan, Xinrong Zhang, Tyler Wong, Ramsey Cheung, Mindie H. Nguyen

**Affiliations:** 1 Division of Gastroenterology and Hepatology, Stanford University Medical Center, Palo Alto, California; 2 Department of Infectious Diseases, The First Affiliated Hospital of Xi’an Jiaotong University, Shaanxi, China; 3 The University of California, Santa Barbara, Santa Barbara; 4 Division of Gastroenterology and Hepatology, Veterans Affairs Palo Alto Healthcare System, Palo Alto, California; 5 Department of Epidemiology and Population Health, Stanford University Medical Center, Palo Alto, California; 6 Stanford Cancer Institute, Stanford University Medical Center, Palo Alto, California

## Abstract

This cohort study examines the association of sex with liver and nonliver adverse events using data from a US nationwide population-based database of patients with metabolic dysfunction–associated steatotic liver disease.

## Introduction

Metabolic dysfunction–associated steatotic liver disease (MASLD) affects approximately 30% of the global population, is increasing, and is a leading cause of liver and nonliver adverse events.^1,2^ However, data on sex differences, particularly with the updated concept of MASLD,^2^ are limited. We investigated the association of sex with liver and nonliver adverse events using data from US patients with MASLD.

## Methods

This cohort study was approved by the institutional review boards of Stanford University and followed the STROBE reporting guideline. We identified adult patients with MASLD from the 2007 to 2022 Merative MarketScan Research Database and used propensity score matching to balance the baseline characteristics of the male and female groups (eMethods, eFigure, and eTable in Supplement 1). The incidence of liver adverse events (cirrhosis, hepatic decompensation, and hepatocellular carcinoma [HCC]) and nonliver adverse events (cardiovascular diseases [CVD], chronic kidney disease [CKD], and nonliver cancer, specifically non–sex-specific cancers) was estimated and compared by sex. Cox proportional hazards regression and restrict mean survival time (RMST) analysis were used to assess the association between sex and the event of interest.

## Results

Of 761 403 patients with MASLD, 344 436 pairs of matched men and women with balanced baseline characteristics were included in the incidence analysis. The mean (SD) age (52.7 [12.4] vs 53.0 [12.3] years), proportions of patients with obesity (16.9% vs 16.8%), diabetes (33.2% vs 33.3%), hypertension (62.6% vs 62.3%), hyperlipidemia (47.7% vs 46.5%), metformin use (10.4% vs 10.4%) or statin use (25.8% vs 25.4%), and mean (SD) Charlson Comorbidity Index (3.91 [2.14] vs 3.91 [1.96]) were all similar between 2 groups.

Women had higher incidence (per 1000 person-years) vs men of any liver adverse event (12.72 vs 11.53) and cirrhosis (12.68 vs 11.55), whereas men had higher incidence of hepatic decompensation (10.40 vs 9.37), HCC (1.88 vs 0.73), CVD (17.89 vs 12.89), CKD (16.61 vs 14.42), and non–sex-specific cancer (6.68 vs 5.06) (Table). The 10-year cumulative incidence rates are shown in the Figure.

**Table.** Incidence Rate of Study Outcomes by Sex and Association of Sex With Study Outcomes in Patients With Metabolic Dysfunction–Associated Steatotic Liver Disease

Outcome	Patients, No.	Person-years, No.	Events, No.	Incidence/1000 person-years	*P* value^a^	HR (95% CI)	*P* value^a^	RMST (95% CI), y	RMST difference (95% CI), y	*P* value^a^
Liver adverse events										
Any liver event										
Female	265 337	986 542	12 548	12.72 (12.50 to 12.94)	<.001	1 [Reference]	<.001	9.41 (9.39 to 9.42)	0.05 (0.04 to 0.07)	<.001
Male	256 443	943 846	10 883	11.53 (11.31 to 11.75)		0.91 (0.88 to 0.92)		9.46 (9.45 to 9.47)		
Cirrhosis										
Female	265 492	987 177	12 516	12.68 (12.46 to 12.90)	<.001	1 [Reference]	<.001	9.41 (9.40 to 9.42)	0.05 (0.04 to 0.07)	<.001
Male	256 720	944 779	10 909	11.55 (11.33 to 11.77)		0.91 (0.89 to 0.93)		9.46 (9.45 to 9.47)		
Hepatic decompensation										
Female	290 663	1 085 347	10 172	9.37 (9.19 to 9.56)	<.001	1 [Reference]	<.001	9.56 (9.55 to 9.57)	−0.04 (−0.05 to −0.03)	<.001
Male	286 799	1 050 649	10 929	10.40 (10.21 to 10.60)		1.11 (1.08 to 1.14)		9.52 (9.51 to 9.53)		
Hepatocellular carcinoma										
Female	303 667	1 138 012	828	0.73 (0.68 to 0.78)	<.001	1 [Reference]	<.001	9.96 (9.96 to 9.97)	−0.06 (−0.06 to −0.05)	<.001
Male	299 809	1 098 329	2068	1.88 (1.80 to 1.97)		2.59 (2.39 to 2.80)		9.91 (9.90 to 9.91)		
Nonliver adverse events										
Cardiovascular disease										
Female	272 124	963 729	12 420	12.89 (12.66 to 13.12)	<.001	1 [Reference]	<.001	9.34 (9.33 to 9.35)	−0.25 (−0.27 to −0.04)	<.001
Male	265 196	910 389	16 286	17.89 (17.62 to 18.17)		1.40 (1.37 to 1.43)		9.08 (9.07 to 9.10)		
Chronic kidney disease										
Female	272 314	955 111	13 770	14.42 (14.18 to 14.66)	<.001	1 [Reference]	<.001	9.29 (9.28 to 9.31)	−0.10 (−0.12 to −0.09)	<.001
Male	266 074	908 301	15 086	16.61 (16.35 to 16.88)		1.16 (1.13 to 1.18)		9.19 (9.18 to 9.20)		
Overall nonliver cancer										
Female	277 066	1 021 598	8925	8.74 (8.56 to 8.92)	.98	1 [Reference]	.99	9.55 (9.54 to 9.56)	0.01 (0.00 to 0.03)	.04
Male	275 249	1 001 053	8743	8.73 (8.55 to 8.92)		1.00 (0.97 to 1.03)		9.56 (9.55 to 9.57)		
Non–sex-specific cancer^b^										
Female	272 948	1 008 948	5107	5.06 (4.92 to 5.20)	<.001	1 [Reference]	<.001	9.73 (9.72 to 9.74)	−0.07 (−0.08 to −0.06)	<.001
Male	272 957	993 822	6634	6.68 (6.52 to 6.84)		1.32 (1.27 to 1.37)		9.66 (9.65 to 9.67)		

Abbreviations: HR, hazard ratio; RMST, restrict mean survival time.

^a^ *P* values were adjusted for multiple testing using the Bonferroni Correction.

^b^ Excludes sex-specific cancers, including breast, ovarian, cervical, uterine, prostate, testicular, and penile cancers.

**Figure.**
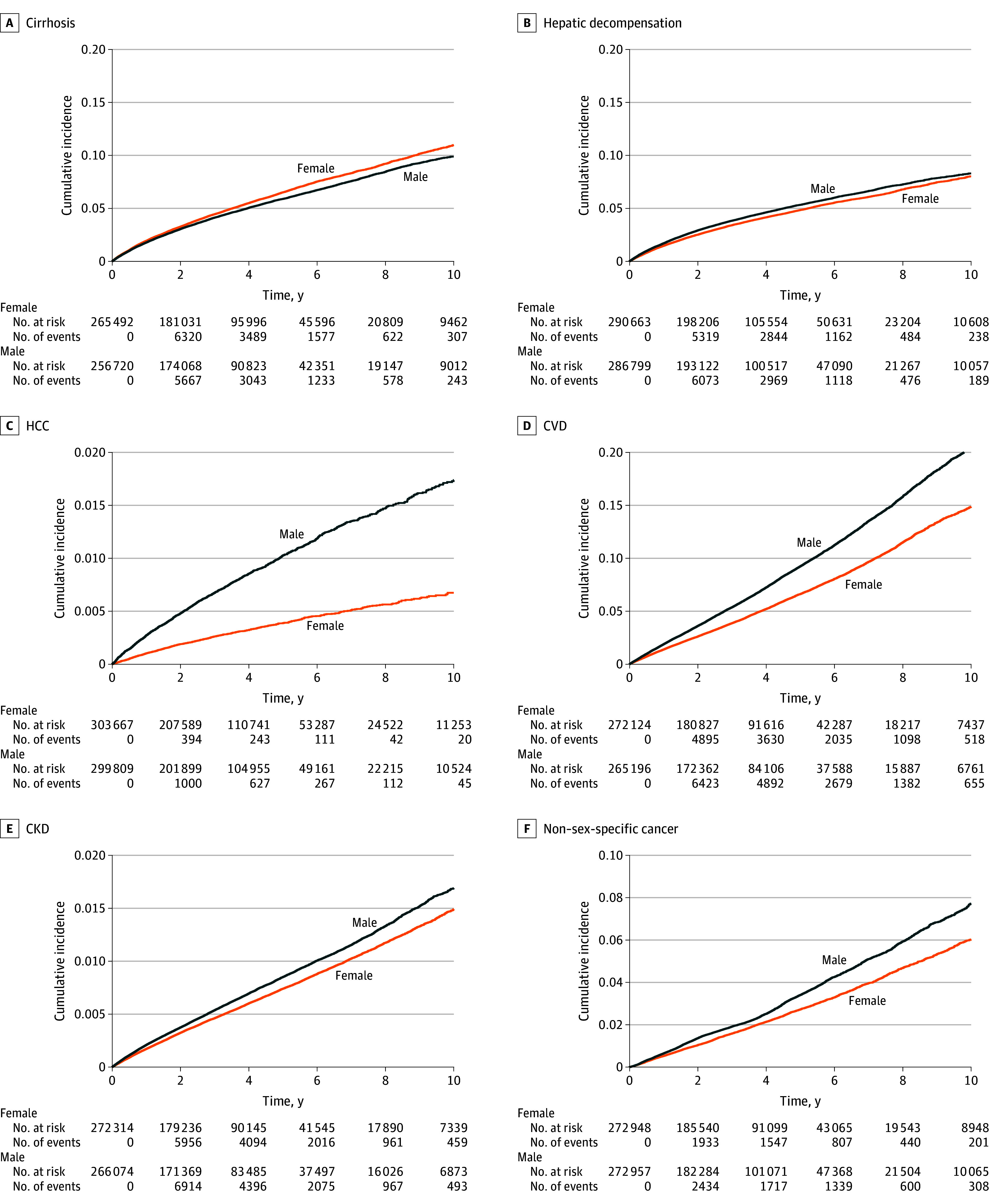
Cumulative Incidence of Liver and Nonliver Adverse Events in Patients With Metabolic Dysfunction–Associated Steatotic Liver Disease by Sex The 10-year cumulative incidences in men and women were 9.9% vs 11.0% for cirrhosis, 8.3% vs 8.0% for hepatic decompensation, 1.7% vs 0.7% for hepatocellular carcinoma (HCC), 20.4% vs 14.9% for cardiovascular disease (CVD), 16.9% vs 14.9% for chronic kidney disease (CKD), and 7.7% vs 6.0% for non–sex-specific cancer. All *P* < .001, adjusted for multiple testing using the Bonferroni correction.

Women had a 9% higher risk of cirrhosis development vs men, and men had an 11% higher risk of hepatic decompensation and more than double the risk of HCC vs women (hazard ratio [HR], 2.59; 95% CI, 2.39-2.80; *P* < .001) (Table). Men also had a 40% higher risk of CVD (HR, 1.40; 95% CI, 1.37-1.43; *P* < .001), a 16% higher risk of CKD (HR, 1.16; 95% CI, 1.13-1.18; *P* < .001), and a 32% higher risk of non–sex-specific cancers (HR, 1.32; 95% CI, 1.27-1.37; *P* < .001) than women (Table). The RMST analysis at the 10-year follow-up showed that women had significantly shorter mean time to the development of cirrhosis, whereas men had significantly shorter mean time to hepatic decompensation, HCC, CVD, CKD and non–sex-specific cancers (Table).

## Discussion

To our knowledge, this is the first and largest cohort study specifically designed to examine the association of sex with adverse clinical outcomes of MASLD. We found higher incidence and risk of cirrhosis in women, consistent with the observation that MASLD is the primary indication for liver transplantation among women in the US.^3^ As in prior studies reporting higher HCC risk in men with other chronic liver diseases vs women,^4^ our study found a significant association between male sex and increased HCC risk among patients with MASLD. In addition, we found higher risks of CVD and non–sex-specific nonliver cancers among male (vs female) patients with MASLD, expanding the existing knowledge of higher CVD and cancer risks for men vs women in the general population.^5,6^ Our study included only patients with private health insurance in the US, so additional studies are needed to examine the association of sex in patients without insurance or with only government-sponsored insurance and patients in other world regions. Our study provides robust evidence of significant sex differences in the risk of both liver and nonliver adverse events in patients with MASLD to support policies for sex-based preventive, monitoring, and therapeutic management strategies of MASLD.

## Notes

Supplement 1.
eMethods.

eReferences
**eFigure.** Study flowchart **eTable.** ICD-9/10-CM diagnosis codes and procedure codes

Supplement 2.
Data Sharing Statement
