# Supplementary material for: Sex Differences in Adverse Liver and Nonliver Outcomes in Steatotic Liver Disease
Source: JAMA Netw Open. 2024 Dec 4;7(12):e2448946. doi: 10.1001/jamanetworkopen.2024.48946 (PMC11618471; doi:10.1001/jamanetworkopen.2024.48946)
Supplement: Supplement 1. — eMethods. eReferences eFigure. Study flowchart eTable. ICD-9/10-CM diagnosis codes and procedure codes [file jamanetwopen-e2448946-s001.pdf]

Supplemental Online Content

Yan T, Zhang X, Wong T, Cheung R, Nguyen MH. Sex differences in adverse liver and nonliver outcomes in steatotic liver disease. *JAMA Netw Open*. 2024;7(12):e2448946. doi:10.1001/jamanetworkopen.2024.48946

eMethods

eReferences

eFigure. Study flowchart

eTable. ICD-9/10-CM diagnosis codes and procedure codes

This supplemental material has been provided by the authors to give readers additional information about their work.

© 2024 Yan T et al. *JAMA Network Open.*

eMethods

Data source

We conducted a retrospective study of adult patients with metabolic dysfunction-associated steatotic liver disease (MASLD) using the Merative™ MarketScan Research Database housed in the Population Health Science Center at Stanford University, Palo Alto, California[1,2]. The Merative™ MarketScan Research Database is a large national administrative claims database with data on approximately 250 million Americans with private health insurance (with or without Medicare) coverage between January 2007 and December 2022. This study was approved by the Institutional Review Boards of Stanford University, Stanford, California, USA. The IRB waived consent because the data were anonymous, in accordance with 45 CFR §46.

Study design and population

We identified patients with MASLD using the nineth or tenth revision Clinical Modification of International Classification of Diseases (ICD-9/10-CM) codes of fatty liver disease combined with the ICD code of at least one of the cardiometabolic risk factors including obesity, diabetes, hypertension, hyperlipidemia, or use of medications for diabetes, hypertension or hyperlipidemia **(eFigure 1 and eTable 1)**. We excluded patients younger than 18 years and those who had significant alcohol use, which was defined by the diagnosis codes indicating alcohol use disorder and/or clinical complications of alcohol use disorders such as alcohol-related pancreatitis, alcohol withdrawal **(eFigure 1 and eTable 1)**. Baseline characteristics evaluated include demographics, geographic region, insurance type, provider specialty type, presence of cirrhosis and viral hepatitis, Charlson comorbidity index (CCI), use of statins and metformin.

Study outcomes and definitions

We defined the index date as the date of the first MASLD diagnosis. The primary study outcomes were incidence of (1) liver adverse events (cirrhosis, hepatic decompensation and hepatocellular carcinoma [HCC]) and (2) non-liver adverse events (cardiovascular diseases [CVD], chronic kidney disease [CKD] and non-liver cancer). We identified these events using ICD-9/10-CM diagnostic codes of cirrhosis, hepatic decompensation, HCC, CVD, CKD and non-liver cancers **(eTable 1)**. Incident events were defined as the first diagnosis of these events following 6 months of study index or after. Patients with prevalent cases of events of interest defined as events occurring before or within 6 months of MASLD date were excluded in incidence analyses of each event of interest. For non-liver cancer, we further identified the non-sex-specific cancers which were defined as cancers that are not sex-specific such as breast, ovarian, cervical, uterine, prostate, testicular and penile cancers.

Statistical analysis

Continuous variables were reported as mean and standard deviation (SD) if normally distributed or median and interquartile ranges if not. Categorical data were reported as numbers and percentages. Patients were classified into 2 study groups: males and females. Differences between groups were compared using the *t* test or Wilcoxon rank-sum test for continuous variables and chi-squared test for categorical variables. Propensity score matching (PSM) with a caliper of 0.1 for age, geographic region, insurance type, provider specialty type, medications including metformin and statins, and comorbidities including diabetes, obesity, hypertension, hyperlipidemia, CVD, CKD, non-liver cancer, and CCI, were performed to balance the baseline characteristics between the two study groups. Variables with standardized mean difference (SMD) <0.1 was considered to be balanced between the two study groups. Kaplan-Meier method was used to examine the cumulative incidence of an event between sex, with log-rank test for comparison of the two study groups. Cox proportional hazards regression was used to estimate the HR for risk of an event of interest. The between-group differences in time-to-event outcomes were analyzed using restrict mean survival time (RMST) based on the Kaplan-Meier curve. The RMST represents the mean event-free time until a time point and is calculated as the area under the Kaplan-Meier curve. All analyses were conducted using R version 4.1.1 (R Foundation for Statistical Computing, Vienna, Austria). Two-sided tests of significance were used, and $P < 0.05$ was considered statistically significant.

eReferences

1. Stanford Center for Population Health Sciences. (2024). Merative™ Marketscan® Databases (Version 3.1) [Data set]. Redivis. https://doi.org/10.57761/n5v8-0v21
2. Stanford Center for Population Health Sciences. (2024). Merative™ Marketscan® Medicare Supplemental (Version 3.1) [Data set]. Redivis. https://doi.org/10.57761/h9zd-f494

© 2024 Yan T et al. *JAMA Network Open.*

eFigure. Study flowchart.

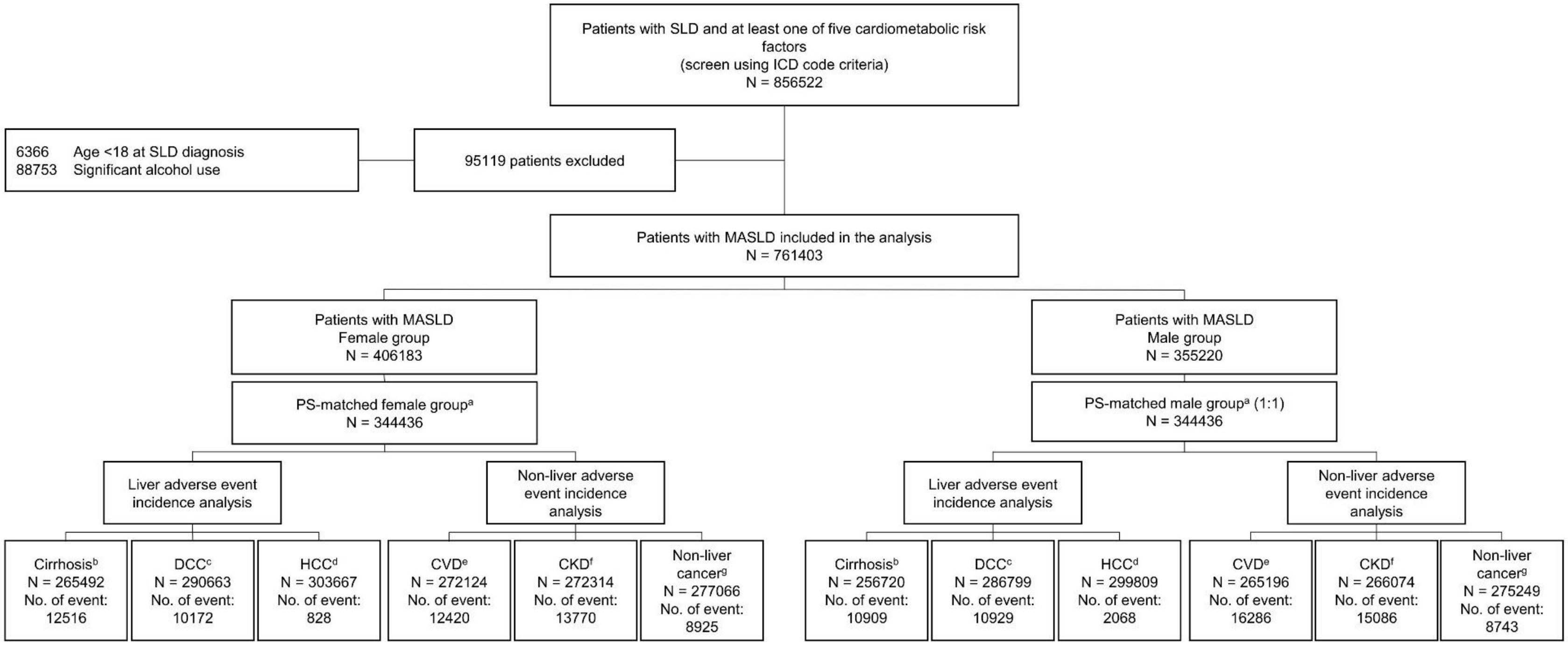

Abbreviations: SLD, Steatotic liver disease; ICD, International Classification of Diseases; MASLD, Metabolic dysfunction-associated steatotic liver disease; PS, Propensity-score; DCC, decompensation; HCC, Hepatocellular carcinoma; CVD, Cardiovascular disease; CKD, Chronic liver disease.

[a]Patients in male and female groups were matched for age, geographic region, insurance type, specialty type, Charlson comorbidity index, drug use, comorbidities including obesity, diabetes, hypertension, hyperlipidemia, CVD, CKD and non-liver cancer. [b-g]Patients with the events ([b]cirrhosis, [c]DCC, [d]HCC, [e]CVD, [f]CKD, [g]non-liver cancer) occurring before or within 6 months of MASLD date were excluded in incidence analyses of each event of interest.

© 2024 Yan T et al. *JAMA Network Open.*

eTable. ICD-9/10-CM diagnosis codes and procedure codes.

Disease	ICD-9/10-CM diagnosis codes/procedure codes
Steatotic liver disease	571.8, 571.9, K76.0, K75.81
Obesity	278.00, 278.01, 278.03, V85.30-V85.39, V85.41-V85.45<br>E66.0, E66.01, E66.09, E66.1, E66.2, E66.8, E66.9<br>Z68.30-Z68.39, Z68.41-Z68.45
Diabetes mellitus	250.0X-250.9X, E10.X-E11.X, E13.X
Hypertension	401, 401.0, 401.1, 401.9, I10
Hyperlipidemia	272.0-272.4, E78.00, E78.1- E78.4, E78.49, E78.5
Significant alcohol use	291, 291.0, 291.1, 291.2, 291.3, 291.4 291.5, 291.8, 291.81, 291.89, 291.9, 303, 303.0, 303.00, 303.01, 303.02, 303.03 303.90, 303.91, 303.92, 303.93, 305, 305.00, 305.01, 305.02, 305.03, 357.5, 425.5, 535.30, 535.31, 571.0, 571.1 571.2, 571.3, 655.4, 760.71, V11.3, E860.0<br>F10.0, F10.1X, F10.2X, F10.9X, G31.2, G72.1, I42.6, K29.20, K29.21, K85.2X, K86.0, Z71.4X, G62.1, E24.4 K70.0-K70.4, K70.9, Q86.0, P04.3, O35.4, O99.31X, T51
Cirrhosis	571.2, 571.5, 572.3, 456.1, 456.21, K70.30, K74.60, K74.69, K76.6, I85.00, I85.10
Hepatic decompensation	
Ascites	789.5, 789.59, R18. 8, K70.31
Spontaneous bacterial peritonitis	567.23, K65.2
Variceal bleeding	456.0, 456.2, 456.8, I85.01, I85.11, I86.4
Hepatic encephalopathy	572.2, K76.82, K72.91, K72.01, K72.11
Hepatorenal syndrome	572.4, K76.7
Hepatocellular carcinoma	155.0, C22.0, C22.8
Cardiovascular diseases	398.91, 402,01, 402.11, 402.91, 404.01, 404.03, 404.11, 404.13, 404.91, 404.93, 425.4-425.9, 428.0, 428.1, 428.20-428.23, 428.30-428.33, 428.40-428.43, 428.9<br>I20.9, I21.0-I21.9, I21.A1, I21.A9, I25, I25.1, I25.10, I25.11X, I25.2, I25.5, I25.84, I25.9, I42.0-I42.9, I43, I50.1-I50.4, I50.8, I50.9
Chronic kidney disease	585.1-585.6, 585.9, 403.00, 403.01, 403.10, 403.11, 403.90, 403.91, 404.00-404.03, 404.10-404.13, 404.90-404.93, V42.0, 996.81, 55.69*<br>N18.1-N18.6, N18.9, I12.0, I12.9, E11.22, E10.22, T86.10, Z94.0, Z48.22
Non-liver cancers	14X.XX-208.XX (excluding 173.XX and 155.0)<br>C00.XX-C97.XX (excluding C43.X, C44.X and C22.0, C22.8)
Sex-specific cancers	
Female-specific cancers	179, 182, 182.0, 182.1, 182.8, C54.0, C54.1, C54.2, C54.3, C54.8, C54.9, C55<br>183, 183.0, C56.1, C56.2, C56.9<br>174, 174.0, 174.1, 174.2, 174.3, 174.4, 174.5, 174.6, 174.8, 174.9, C50.011, C50.012, C50.019, C50.111, C50.112, C50.119, C50.211, C50.212, C50.219, C50.311, C50.312, C50.319, C50.411, C50.412, C50.419, C50.511, C50.512, C50.519, C50.611, C50.612, C50.619, C50.811, C50.812, C50.819, C50.911, C50.912, C50.919
Male-specific cancers	185, C61<br>186, 186.0, 186.9, C62, C62.0, C62.00, C62.01, C62.02, C62.1, C62.10, C62.11, C62.12, C62.9, C62.90, C62.91, C62.92

© 2024 Yan T et al. *JAMA Network Open.*

	187, 187.1, 187.2, 187.3, 187.4, 187.5, 187.6, 187.7, 187.8, 187.9, C60, C60.0, C60.1, C60.2, C60.8, C60.9 C63, C63.0, C63.00, C63.01, C63.02, C63.1, C63.10, C63.11, C63.12, C63.2, C63.7, C63.8, C63.9

Abbreviations: ICD-9/10-CM, International Classification of Diseases, Ninth/Tenth Revision, Clinical Modification.
*Procedure code.

© 2024 Yan T et al. *JAMA Network Open.*
