# Supplementary material for: Sex Differences in Adverse Liver and Nonliver Outcomes in Steatotic Liver Disease
Source: JAMA Netw Open. 2024 Dec 4;7(12):e2448946. doi: 10.1001/jamanetworkopen.2024.48946 (PMC11618471; doi:10.1001/jamanetworkopen.2024.48946)
Supplement: Supplement 2. — Data Sharing Statement [file jamanetwopen-e2448946-s002.pdf]

Data Sharing Statement

Yan. Sex Differences in Adverse Liver and Nonliver Outcomes in Steatotic Liver Disease.
JAMA Netw Open. Published December 04, 2024. doi:10.1001/jamanetworkopen.2024.48946

Data
Data available: No
